# Detrimental Effects of HMGB-1 Require Microglial-Astroglial Interaction: Implications for the Status Epilepticus -Induced Neuroinflammation

**DOI:** 10.3389/fncel.2019.00380

**Published:** 2019-08-27

**Authors:** Gerardo Rosciszewski, Vanesa Cadena, Jerónimo Auzmendi, María Belén Cieri, Jerónimo Lukin, Alicia R. Rossi, Veronica Murta, Alejandro Villarreal, Analia Reinés, Flávia C. A. Gomes, Alberto Javier Ramos

**Affiliations:** ^1^Laboratorio de Neuropatología Molecular, Instituto de Biología Celular y Neurociencia “Prof. E. De Robertis” UBA-CONICET, Facultad de Medicina, Universidad de Buenos Aires, Buenos Aires, Argentina; ^2^Laboratorio de Neurofarmacología, Instituto de Biología Celular y Neurociencia “Prof. E. De Robertis” UBA-CONICET, Facultad de Medicina, Universidad de Buenos Aires, Buenos Aires, Argentina; ^3^Instituto de Ciências Biomédicas, Universidade Federal do Rio de Janeiro, Rio de Janeiro, Brazil

**Keywords:** epilepsy, glia, inflammation, seizures, neuronal death

## Abstract

Temporal Lobe Epilepsy (TLE) is the most common form of human epilepsy and available treatments with antiepileptic drugs are not disease-modifying therapies. The neuroinflammation, neuronal death and exacerbated plasticity that occur during the silent period, following the initial precipitating event (IPE), seem to be crucial for epileptogenesis. Damage Associated Molecular Patterns (DAMP) such as HMGB-1, are released early during this period concomitantly with a phenomenon of reactive gliosis and neurodegeneration. Here, using a combination of primary neuronal and glial cell cultures, we show that exposure to HMGB-1 induces dendrite loss and neurodegeneration in a glial-dependent manner. In glial cells, loss of function studies showed that HMGB-1 exposure induces NF-κB activation by engaging a signaling pathway that involves TLR2, TLR4, and RAGE. In the absence of glial cells, HMGB-1 failed to induce neurodegeneration of primary cultured cortical neurons. Moreover, purified astrocytes were unable to fully respond to HMGB-1 with NF-κB activation and required microglial cooperation. In agreement, *in vivo* HMGB-1 blockage with glycyrrhizin, immediately after pilocarpine-induced status epilepticus (SE), reduced neuronal degeneration, reactive astrogliosis and microgliosis in the long term. We conclude that microglial-astroglial cooperation is required for astrocytes to respond to HMGB-1 and to induce neurodegeneration. Disruption of this HMGB-1 mediated signaling pathway shows beneficial effects by reducing neuroinflammation and neurodegeneration after SE. Thus, early treatment strategies during the latency period aimed at blocking downstream signaling pathways activated by HMGB-1 are likely to have a significant effect in the neuroinflammation and neurodegeneration that are proposed as key factors in epileptogenesis.

## Introduction

Epilepsy is a devastating neurological disease characterized by recurrent seizures. A significant number of patients develop refractory epilepsy that is unresponsive to currently available treatments, which leaves them with very limited clinical options, being surgical resection of the epileptic focus the most common choice. Retrospective studies have shown that most patients suffering of Temporal Lobe Epilepsy (TLE), the most common human epilepsy, refer an initial precipitating event (IPE) in early childhood, followed by a silent period when the epileptic seizures begin (Cendes et al., 1993; French et al., 1993; Hamati-Haddad and Abou-Khalil, 1998; Theodore et al., 1999; Blume, 2006; Majores et al., 2007). Different types of IPE have been described, but complex febrile seizures with status epilepticus (SE) are frequently associated with adult TLE (Cendes et al., 1993; French et al., 1993; Koyama et al., 2012). The pilocarpine experimental model of TLE reproduces in rodents most features of human TLE, including the IPE induced by acute SE and a latency period before the onset of spontaneous seizures (reviewed in Curia et al., 2008). Previous studies performed in our laboratory, during the latency period that follows pilocarpine-induced seizures, have shown early neuroinflammation, macrophage infiltration and exacerbated neuronal plasticity, which seem to have a main role in the epileptogenesis (Rossi et al., 2013, 2017).

In general terms, acute brain injury causes the release of Damage Associated Molecular Patterns (DAMP). DAMP are intracellular molecules capable of activating the Pattern Recognition Receptors (PRR) such as Toll-Like Receptors (TLR) and the Receptor for Advanced Glycation End products (RAGE). The result of this interaction is the activation of downstream signaling cascades in immune-competent cells eventually leading to the expression of multiple pro-inflammatory mediators, mainly in an NF-κB -dependent manner. High Mobility Group Box 1 (HMGB1) is an ubiquitous nuclear protein that is implicated in the maintenance of chromatin structure (Ellwood et al., 2000; Verrijdt et al., 2002) and that, following Central Nervous System (CNS) injury, is released as a DAMP (Qiu et al., 2008; Maroso et al., 2010). The release of HMGB-1 has been demonstrated in human patients (reviewed in Parker et al., 2017) and in different models of experimental brain injury, including epileptic seizures produced by different experimental paradigms (Maroso et al., 2010); ischemia by middle cerebral artery occlusion (Qiu et al., 2008); traumatic brain injury (TBI) (Laird et al., 2014; Braun et al., 2017); cortical spreading depression (Takizawa et al., 2017); intracerebral hemorrhage (Wang et al., 2017) and it was shown to be increased in aging (Fonken et al., 2016). Moreover, several recent reports have proposed that HMGB-1 release is involved in the epileptogenic process that ultimately develops into overt disease (Fu et al., 2017; Walker et al., 2017a, b; Yang et al., 2017).

The abundant evidence in this regard has raised the hypothesis that blockage of HMGB-1 may blunt the pro-inflammatory activation of PRR that follows brain injury (Yang et al., 2010). In line with this idea, a number of reports have shown that HMGB-1 blockage reduces inflammation and improves behavioral recovery in experimental stroke (Wang C. et al., 2016).

In this work, we investigated the HMGB-1 effects on neuronal survival, and specifically, how glial cells interact to induce neuronal alterations and analyzed the signaling pathways driving those effects. Having in mind that HMGB-1 is released after SE, we aimed to block HMGB-1 signaling pathway with glycyrrhizin after pilocarpine-induced SE. Our results showed that HMGB-1 exposure induces reactive gliosis in astrocytes and microglia, and exerts glia-mediated detrimental effects on neurons. The NF-κB signaling pathway was activated by HMGB-1 by engaging TLR2, TLR4 and RAGE innate immunity receptors. In order to fully activate glial NF-κB signaling, HMGB-1 requires the cooperation between microglia and astrocytes. Finally, we observed that HMGB-1 blockage with glycyrrhizin *in vivo* immediately after pilocarpine-induced seizures reduces neuronal degeneration and reactive gliosis in the long term. Taken together, our results show that HMGB-1 has distinct effects on the different CNS cell types, in the context of the early stages following a typical acute precipitating injury in epilepsy. Thus, early blockage of HMGB-1 is likely to have a beneficial effect, as it would blunt pro-inflammatory cooperation between astrocytes and microglia during a critical period following seizures-induced IPE, a key event related to epileptogenesis.

## Materials and Methods

Cell culture reagents were obtained from Invitrogen Life Technologies (Carlsbad, United States). Fetal calf serum (FCS) was purchased from Natocor (Córdoba, Argentina). Antibodies were purchased from Chemicon-Millipore (mouse monoclonal anti-Actin, cat# MAB1501; mouse monoclonal anti-NeuN, cat# MAB 377; rabbit polyclonal anti-MAP-2, cat# AB5622), Sigma (mouse monoclonal anti-S100B cat# S2532; mouse monoclonal anti-Glial Fibrillary Acidic Protein, GFAP cat# G3893), Santa Cruz (rabbit polyclonal anti-TREM-2 cat# SC-48765; rabbit polyclonal anti-p65 cat# SC-372), Abcam (goat polyclonal anti-Iba-1, cat# ab5076); Dako (rabbit polyclonal anti-GFAP, cat# Z0334), and Promega (mouse monoclonal anti-β-3-tubulin, cat# G712A). Poly-L-lysine, DAPI (4′,6-diamidino-2-phenylindole); glycyrrhizin, human recombinant HMGB1 and other chemicals were from Sigma (United States). Fluorescent secondary antibodies and peroxidase conjugated secondary antibodies were purchased from Jackson Immunoresearch (United States).

### Animals and Lithium-Pilocarpine Model of TLE

Adult male Wistar rats (250–300 g) were obtained from the Animal Facility of the School of Exact and Natural Sciences, University of Buenos Aires. TLR4 (TLR4 B6.B10ScN-*Tlr4*^*lps–del*^/JthJ) and TLR2 (B6.129-*Tlr2*^*tm*1*Kir*^/J) knockout mice (The Jackson Laboratory, United States) were kindly provided by Dr. P. Iribarren and Dr. M. Maccioni (CIBICI, UNC, Córdoba, Argentina). Animals were housed in a controlled environment (12/12-h light/dark cycle, controlled humidity and temperature, free access to standard laboratory food and water) under the permanent supervision of professional technicians.

All procedures involving animals and their care were conducted in accordance with our institutional guidelines, which comply with the NIH guidelines for the Care and Use of Laboratory Animals, the principles presented in the Guidelines for the Use of Animals in Neuroscience Research by the Society for Neuroscience and the ARRIVE guidelines, and were approved by the CICUAL committee of the School of Medicine, University of Buenos Aires (Res. Nr. 1278/2012). All efforts were made to minimize animal suffering and to reduce the number of animals used.

Rats were randomly assigned to the different treatments groups and subjected to the lithium-pilocarpine model of TLE as described in Rossi et al. (2013). Briefly, animals were intraperitoneally (i.p.) injected with 3 mEq/kg lithium chloride (LiCl) or saline and 20 h later received either i.p. saline or 30 mg/kg pilocarpine (Li-pilo group). Animals that received saline-saline (control group) or Li-saline did not show significant differences either in behavioral or morphometric parameters. The development of behavioral seizures was evaluated according to the Racine scale (Racine et al., 1972) and SE was defined as continuous seizures with a Racine score of 3 to 5, without returning to lower stages for at least 5 min. Approximately 70% of the pilocarpine treated rats showed acute behavioral features of SE between 40 and 60 min after pilocarpine injection (SE group). Thirty percent of the animals that were injected with pilocarpine did not develop SE, showing only behavioral signs corresponding to stages 1–2 of the Racine score and were not used for this study. All animals received 20 mg/kg diazepam 20 min after the onset of SE and doses were repeated as needed to terminate SE. Half of the animals that develop SE received glycyrrhizin i.p. at a dose of 333 mg/kg every 12 h during 4 days. Fifteen days post-SE (DPSE), animals were deeply anesthetized with ketamine/xylazine (90/10 mg/kg, i.p.) and fixed by intracardiac perfusion through the left ventricle. Dissected brains were cryoprotected, snap frozen and coronal 30 μm thick brain sections were cut using a cryostat as previously described (Aviles-Reyes et al., 2010). Free floating sections were kept in a cryoprotective solution (30% glycerol, 20% ethylene glycol in 0.05 M phosphate buffer) at −20°C until use.

### Cell Culture

Rat or mice glial cell cultures were performed using the same protocol. Brains from neonatal rats, wild type mice or transgenic mice pups (3 days old) were removed and brain cortices were isolated following the procedure previously described (Villarreal et al., 2014). When mixed glial cultures reached confluence (typically 8–10 days), they were collected following trypsin treatment and seeded for the experimental procedures. Hippocampal neuro-glial cultures (containing glia and neurons), primary cortical neurons, cortical mixed glial cultures (containing approximately 60% astrocytes, 40% microglia), cortical astroglial enriched cultures (99% astrocytes) or microglial cultures (>99% microgliocytes) were obtained as described in Rosciszewski et al. (2018). For immunocytochemistry, primary cell cultures were washed with cold PBS and fixed with 4% paraformaldehyde plus 4% sucrose in PBS pH 7.2 for 15 min at room temperature. The procedure was then followed as stated below. For cell viability analysis, cell survival was measured by the MTT [(3-(4,5-dimethylthiazol-2-yl)-2,5-diphenyltetrazolium bromide)] assay (Mosmann, 1983) with some modifications (Alaimo et al., 2011). Briefly, MTT was added to each well (0.125 mg/ml) and incubated for 2 h at 37°C. Then, formazan reaction product was solubilized in DMSO and absorbance was measured at 570 nm with background subtraction at 655 nm in a microplate reader (BIO-RAD Laboratories, Hercules, CA, United States). The MTT reduction activity was expressed as the absorbance at 570 nm.

Astrocyte conditioned medium (ACM) was prepared as described previously (Diniz et al., 2012). Briefly, confluent astroglial-enriched cultures were washed to eliminate residual FCS and then exposed to 500 ng/ml HMGB-1 for 24 h in serum free medium. Then, cells were washed and cultures were maintained 24 h in serum-free medium. Finally, ACM was centrifuged to remove cellular debris and kept at −80°C until use.

For neuron-glial reconstituted cultures, primary cortical neurons and glial cells were grown separately as stated above and we designed a two-chamber system to allow co-culture without cell contact. Neurons were grown at a density of 7 × 10^3^ cells/cm^2^ on poly-L-lysine-coated glass coverslips, and maintained for 10 days in NeuroBasal medium (Invitrogen) supplemented with 2% B27 (Invitrogen) and 0.5 mM glutamine. Glial cells were grown in 12-well plastic plates for 7 days and exposed to HMGB-1 500 ng/ml or LPS 25 ng/ml for 3 h. Glial cells were washed and medium was replaced by a 1:1 mixture of supplemented Neurobasal and DMEM culture medium. Thereafter, a coverslip containing 10 DIV (DIV, days *in vitro*) primary cortical neurons was placed on top of the glial culture supported by U-shape custom-made sterile surgical steel spacers. The coverslips were placed with neurons facing the glial cell layer and the tissue culture medium covered all the system. Plates containing the co-culture were incubated for additional 24 h and then cells were fixed separately to analyze neuronal survival.

### RT-PCR Assays

RT-PCR assays were performed as previously described (Rosciszewski et al., 2018). Briefly, RNA was isolated using the RNAeasy Mini kit (Qiagen, Germany). The cDNA was generated using the Omniscript RT kit (Qiagen) with random hexamers (Roche Products, United States). PCR were performed using specific primers: Actin (Fwd: CAC CAC TTT CTA CAA TGA GC; Rev: CGG TCA GGA TCT TCA TGA GG; amplification product: 323 bp); TLR4 (Fwd: GCC GGA AAG TTA TTG TGG TGG T; Rev: ATG GGT TTT AGG CGC AGA GTT T; amplification product: 356 bp). Both TLR4 and actin were amplified by 35 cycles and annealing temperature was 58°C for both genes. PCR products were run in a 1.5% agarose gel and imaged using a VersaDoc 4000 imaging system (Bio-Rad, United States). Each experiment included negative controls in which Omniscript reactions were performed in the absence of reverse transcriptase. All samples were run in triplicate. Detailed PCR protocols are available from authors under request.

### Immunohistochemistry and Immunofluorescence

Brain sections from control and treated animals were simultaneously processed in free-floating state and immunolabelling was detected with diaminobenzidine (DAB) as described previously (Aviles-Reyes et al., 2010; Angelo et al., 2014). For immunofluorescence studies on tissue sections, primary and secondary antibodies were diluted in a solution containing 3% normal horse serum and 1% Triton X-100 in PBS. Isotypic specific secondary antibodies were labeled with Alexa 488 or Alexa 594. Counterstaining was performed with 0.1 μg/ml DAPI. For cell cultures, fixed cells were washed three times with cold PBS and permeabilized with 0.1% Triton X-100. The staining procedure was identical as above except that Triton X-100 was not included in blocking and antibody solutions. Epifluorescence images were obtained using an Olympus IX-81 microscope equipped with a DP71 camera (Olympus, Japan); or a Zeiss Axiophot (Carl Zeiss, Germany) microscope equipped with a Q5 digital camera (Olympus, Japan).

### Quantitative Studies and Statistical Analysis

Changes in astroglial cell morphology as well as neuronal or glial cell counting were evaluated using the NIH ImageJ software on cells observed with phase contrast or immunostained as mentioned in figure legends. Cell counting was performed with ImageJ Cell Counter plugin. Neurite length and area covered by immunostaining was also done with ImageJ software as previously described (Angelo et al., 2014). Cell viability was analyzed biochemically with MTT assay, as described above, or by observing the nuclear morphology with DAPI as indicated in figure legends. Observers were blinded with respect to the experimental conditions. *In vitro* experiments were run in triplicates, a minimum of ten photographs were taken in each well of the triplicates and experiments were repeated three times. *In vivo* experiments were done with six animals per group and only control animals or those pilocarpine-treated that developed SE were used for glycyrrhizin administration. A minimum of 10 tissue sections per animal were used for each morphometrical analysis. Data were analyzed for normal distribution and homogeneity of variances and subjected to appropriate parametric or non-parametric statistical tests as specified in figure legends. Statistical analyses were performed using GraphPad Prism 5.0 (GraphPad Software, United States) and statistical significance was assumed when *p* < 0.05.

## Results

### HMGB-1 Exposure Induces Reactive Gliosis and Dendrite Loss in Hippocampal Neuro-Glial Mixed Culture

**FIGURE 1:**
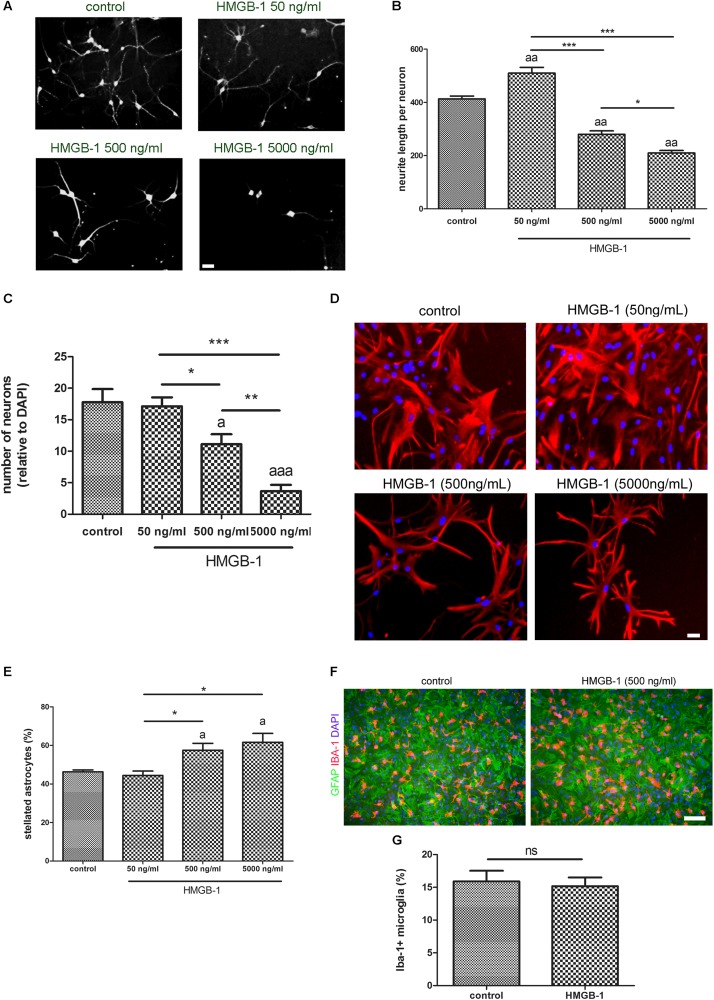
HMGB-1 effects on hippocampal neuro-glial mixed cultures. Rat hippocampal mixed cultures (10–12 DIV) containing neurons and glial cell types were exposed to HMGB-1 for 24 h. **(A)** Representative images of hippocampal neuron morphology in the mixed culture identified by beta-3-tubulin immunostaining; bar = 20 μm. **(B)** Quantitative analysis of neurites evaluated as the total length of neurites per neuron. **(C)** Quantitative analysis of the surviving neurons after 24 h of exposure to HMGB-1. The number of neurons per field was represented as the ratio of neurons vs. the total number of DAPI + nucleus per field. **(D)** Representative images of GFAP-immunostained astrocytes in hippocampal mixed cultures exposed to HMGB-1; bar = 15 μm. **(E)** Quantitative analysis of the stellated reactive GFAP + astrocytes abundance in hippocampal mixed cultures. Stellated fibrilar GFAP + astrocytes were counted with the ImageJ plugin Cell Counter and expressed as the percent of GFAP + cells in each field. **(F)** Representative images of Iba-1 + microgliocytes in hippocampal mixed cultures co-stained with GFAP astrocytic cell marker; bar = 100 μm. **(G)** Quantitative analysis of the microgliocytes cell abundance in the hippocampal mixed cultures showing that microglial abundance is not significantly affected by HMGB-1 exposure. Statistical analyses were performed by one way ANOVA and Student Newman–Keuls post-test, with statistical significance represented as ^∗^*p* < 0.05, ^∗∗^*p* < 0.01, and ^∗∗∗^*p* < 0.001. In multiple comparisons, statistical significance against control group is shown as “a”; “aa” and “aaa” that represents *p* < 0.05, *p* < 0.01, or *p* < 0.001, respectively. Data presented in the graphs are mean ± SEM from three experiments.

Primary hippocampal mixed cultures containing neurons and glia were exposed to increasing concentrations of recombinant HMGB-1: 50 ng/ml, 500 ng/ml, and 5000 ng/ml for 24 h. As shown in Figures 1A,B, neurons from the neuro-glial culture showed an increase in dendrite length at low 50 ng/ml HMGB-1 and then a dose-dependent reduction in the dendrite length at higher concentrations (500–5000 ng/ml) reaching a significant neurodegenerative toxic effect at 5000 ng/ml. In fact, the relative number of neurons in the mixed culture was dose-dependently reduced after exposure to higher doses of HMGB-1 (Figure 1C). An analysis of astroglial cell population in the culture showed that 24 h exposure to HMGB-1 induced astroglial stellation at 500 and 5000 ng/ml HMGB-1 (Figures 1D,E). The observation of glial pyknotic cell nuclei at 5000 ng/ml dose precluded further use of this high dose in the next experiments due to toxic effects for astrocytes. Microglial cell population was present in the hippocampal mixed culture as shown in Figure 1F, however, HMGB-1 exposure did not significantly altered the microglial cell abundance (Figure 1G). Having in mind that astroglial stellation is considered the *in vitro* correlation of reactive gliosis, we conclude that exposure to high HMGB-1 levels induces reactive astrogliosis, dendrite loss and neuronal degeneration in mixed neuro-glial hippocampal cultures.

### HMGB-1 Exposure Does Not Affect Neurons in the Absence of Glial Cells

**FIGURE 2:**
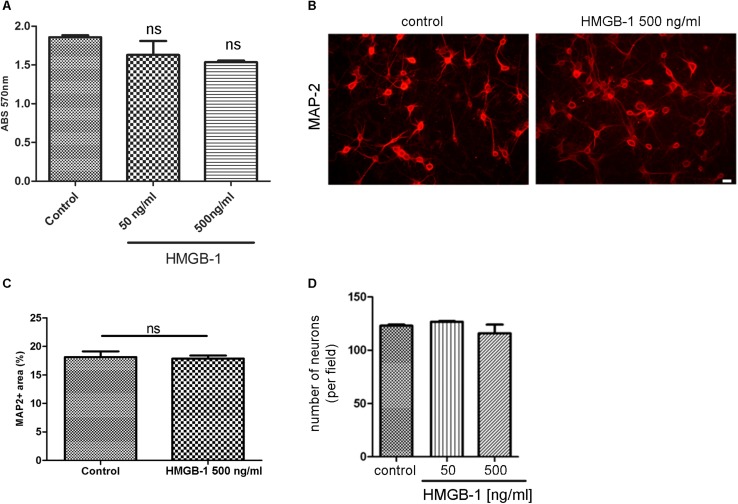
HMGB-1 effects in primary neuronal cultures. **(A)** Quantitative analysis of rat primary cortical neuron survival exposed to HMGB-1 during 24 h and analyzed by the MTT survival assay with results shown as absorbance at 570 nm. **(B)** Representative images of rat primary cortical neurons exposed to HMGB-1 (500 ng/ml) for 24 h and immunostained for MAP-2; bar = 10 μm. **(C)** Quantitative analysis of the area occupied by MAP-2 + neurons after exposure to 500 ng/ml HMGB-1. **(D)** Number of neurons showing normal nuclei per microscopic field analyzed by MAP-2 and DAPI co-staining after 24 h of exposure to HMGB-1 as indicated. Statistical analyses were performed by one way ANOVA. Data presented in the graphs are mean ± SEM from three experiments.

We then tested the effect of HMGB-1 on primary neuronal cultures in the absence of glial cells. For that purpose, primary cortical neurons were exposed to HMGB-1 and neuronal survival was assessed by the MTT survival assay. As shown in Figure 2A, neuronal survival was not significantly affected by exposure to neither 50 nor 500 ng/ml HMGB-1. Primary cortical neurons morphology and proximal dendritic trees stained for the dendritic marker MAP-2 were also evaluated in these cultures and they were not significantly affected by HMGB-1 exposure (Figure 2B). Quantitative evaluation of MAP-2 + areas in primary cortical neurons is a sensitive parameter that detects not only changes in somatic MAP-2 expression, but also alterations in dendrite morphology and complexity. Following HMGB-1 exposure, this parameter did not show statistical differences between control and HMGB-1-exposed neurons (Figure 2C). In addition, there was not a significant neuronal loss in HMGB-1-exposed primary neuronal cultures (Figure 2D). We conclude that direct contact with HMGB-1 does not affect neuronal survival in absence of glial cells.

### HMGB-1 Neurodegenerative Effects Are Mediated by Glial Cells

**FIGURE 3:**
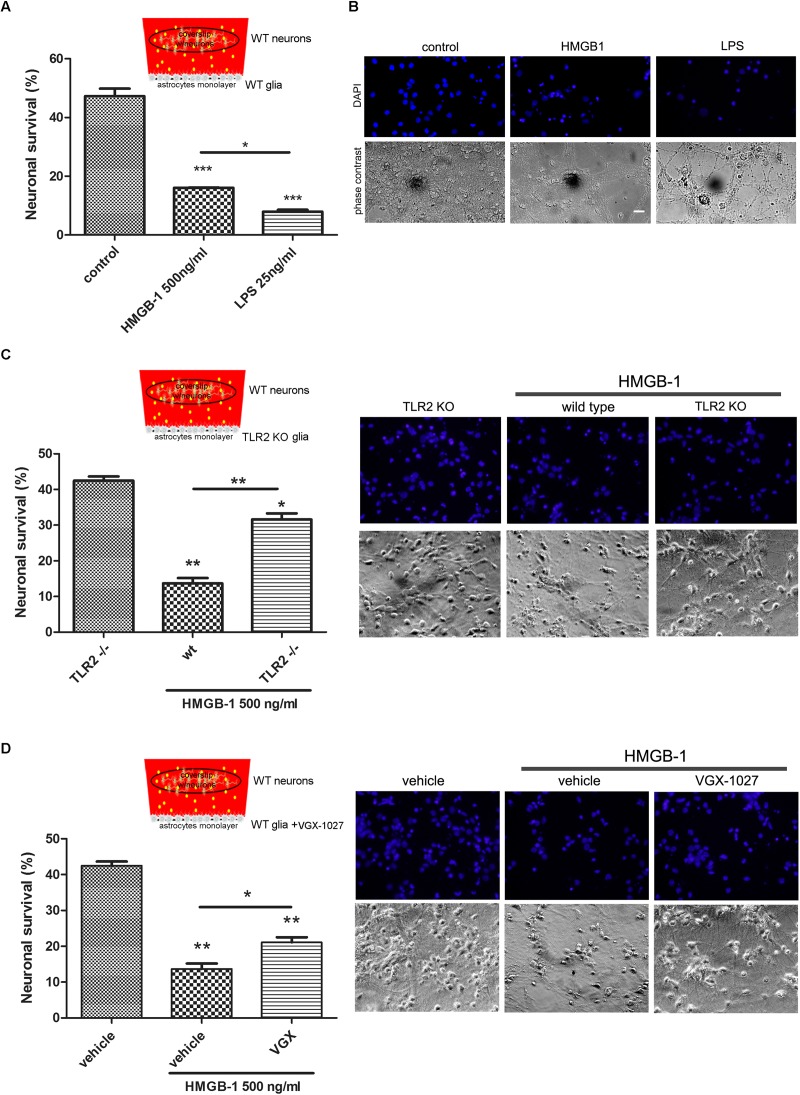
Pattern Recognition Receptor (PRR) requirement for HMGB-1 effects in neuronal survival. Mice primary cortical neuronal and glial cultures were allowed to grow separately. Glia was exposed to HMGB-1 or LPS during 3 h, medium was replaced and then glia and primary cortical neurons (10DIV) were co-cultured without physical contact but sharing the same culture medium for 24 h to study neuronal survival. **(A)** Quantitative analysis of neuronal survival analyzed by counting homogeneous, non-pycnotic, DAPI-stained neuronal nuclei per field. **(B)** Representative images of primary cortical neurons after co-culture with glial cells exposed to HMGB-1. Bar = 30 μm. **(C,D)** Quantitative analysis of neuronal survival in co-cultures performed as described above but using TLR2 knockout glia or VGX-1027 (TLR4 signaling inhibitor)-treated glia (10 μg/ml; 1 h preincubation). Statistical analyses were performed by one way ANOVA and Student Newman–Keuls post-test, with statistical significance is represented as ^∗^*p* < 0.05, ^∗∗^*p* < 0.01, and ^∗∗∗^*p* < 0.001. Data presented in the graphs are mean ± SEM from three experiments.

Having established that HMGB-1 exposure induces neuronal alterations in neuro-glial mixed cultures, and that direct contact with HMGB-1 does not induce *in vitro* neurodegeneration, we then wondered whether glial-derived factors released by HMGB-1-activated glia could alter neuronal survival in culture. In order to answer this question, we performed glio-neuronal reconstituted co-cultures in a modified two chamber design, which prevents glial-neuronal cell contact, but allows diffusion of soluble molecules among the cells. Mixed glial cultures were seeded separately from neurons and exposed to HMGB-1 or LPS as a positive pro-inflammatory-neurodegenerative stimulus for 3 h. Then, medium was replaced by a 1:1 DMEM/neurobasal mixture and a coverslip with primary cortical neurons was added to the glial culture for 24 h to evaluate neuronal survival. As shown in Figures 3A,B, neuronal survival was significantly reduced by HMGB-1-treated glia, but to a lesser extent than in LPS-exposed glial cells. Considering that HMGB-1 effects are proposed to be mediated by PRR, mainly TLR2 and TLR4, we then performed the same experimental design but using primary glia from TLR2 knockout mice, or exposed to LPS and the antagonist VGX-1027 to block TLR4. As expected, HMBG-1 was less efficient in inducing neuronal death in glial cultures obtained from TLR2 knockout animals or exposed to TLR4 blockade (Figures 3C,D). We conclude that detrimental HMGB-1 effects on neurons are mediated by glial cells by engaging TLR2- and TLR4-signaling.

### HMGB-1 Induces TLR4 Expression and Activates NF-κB Dependent Signaling in Primary Astroglial Cultures Containing Microglia

**FIGURE 4:**
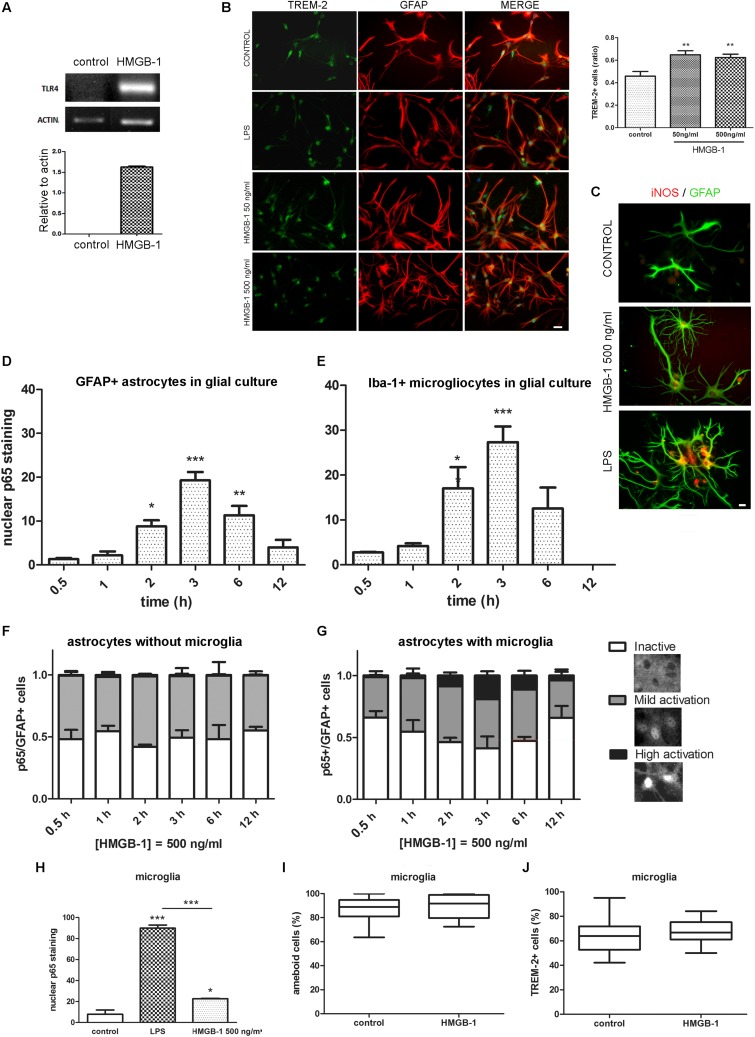
Differential HMGB-1 effects in astrocytes and microglia. **(A)** End point RT-PCR of rat glial mixed cultures containing astrocytes and microglia were exposed to 500 ng/ml HMGB-1 for 18 h to evaluate TLR4 mRNA expression; Actin was used as loading control. **(B)** Rat mixed glial cultures were exposed to HMGB-1 (50 or 500 ng/ml) or 25 ng/ml LPS and TREM-2 expression was evaluated after 24 h; bar = 20 μm. The graph shows the ratio of TREM-2 + cells in culture relative to the total number of Hoechst + stained cells. **(C)** Representative images of iNOS/GFAP immunostaining showing the differential effects in astroglial morphology and iNOS expression of HMGB-1 and LPS exposure on mixed glial cell cultures after 24 h of exposure; bar = 10 μm. **(D)** Quantitative analysis of p65 nuclear localization over time as the percentage of GFAP + astrocytes from mixed glial cultures showing nuclear p65 staining (NF-κB activation) after 500 ng/ml HMGB-1 exposure. **(E)** A similar experiment in mixed glial culture showing the Iba-1 + microglia with nuclear p65 staining over time after 500 ng/ml HMGB-1 exposure. **(F,G)** Astrocyte enriched cultures (less that 1% microglia) **(F)** or mixed glia **(G)** showing the percentage of GFAP + astrocytes with different patterns of NF-κB p65 subunit localization at different time points after 500 ng/ml HMGB-1 exposure. High activation was defined as predominant nuclear p65; mild activation is nuclear and cytoplasmic p65 of equivalent intensity, and inactive is cytoplasmic-only p65 immunostaining. Insets represent typical patterns of the NF-κB p65 subunit localization in astrocytes. Note the absence of cells showing highly activated NF-κB in astroglial-enriched culture. **(H)** Rat microglial culture (> 99% Iba-1 + microglia) showing the p65 nuclear localization after 3 h exposure to 25 ng/ml LPS (pro-inflammatory control) or 500 ng/ml HMGB-1. **(I,J)** Rat microglial cultures were exposed to 500 ng/ml HMGB-1 for 18 h and the percentage of amoeboid microglia **(I)** or TREM-2 immunostained microgliocytes **(J)** were evaluated. Statistical analyses were performed by one way ANOVA and Student Newman–Keuls post-test, with statistical significance represented as ^∗^*p* < 0.05, ^∗∗^*p* < 0.01, and ^∗∗∗^*p* < 0.001. Data presented in the graphs are mean ± SEM from three experiments. In **(I,J)**, Mann–Whitney non-parametrical test was used and data were represented as the median with box showing the interquartile range and whiskers showing the highest and lowest values.

In different cell types, including glia, engagement of TLR2 and TLR4 classically activates feed-forward loops that stimulate innate immunity receptor expression and pro-inflammatory polarization by stimulating downstream NF-κB signaling. To evaluate if HMGB-1 was able to induce these effects, mixed glial cultures containing astrocytes and microglia were exposed to HMGB-1, and TLR4, and TREM-2 innate immunity receptor expression was assessed. As shown in Figure 4A, HMGB-1 exposure increased TLR4 mRNA expression in glial cells. Immunocytochemistry experiments also showed that HMGB-1 increased TREM-2 (Figure 4B) and augmented iNOS expression in glial cells, although to a lesser extent than the classical pro-inflammatory molecule LPS (Figure 4C). TREM-2 expression was mainly regarded to myeloid-derived cells and microglia, but some groups, including ours have shown that TREM-2 can be expressed by reactive astrocytes (Rosciszewski et al., 2018). In addition, HMGB-1 induced morphological changes in GFAP + astrocytes toward a stellated and highly ramified reactive phenotype (Figure 4C). We then looked into NF-κB activation, which is the canonical TLR2 and TLR4 downstream pathway. Analysis of NF-κB p65 subunit nuclear localization in glial mixed cultures, containing astrocytes and microglia, showed that HMGB-1 exposure induces a time-dependent NF-κB activation in both cell types, identified with GFAP and Iba-1, respectively (Figures 4D,E). However, a closer cell-type specific analysis in these glial mixed cultures revealed a higher microglial NF-κB activation following HMGB-1 treatment (Figures 4D,E). Then, we compared the results found in mixed glial cultures with experiments in astroglial enriched cultures obtained by reducing the amount of microgliocytes to less than 1% by shacking and subsequent 5-fluorouracyl treatment, as described in Rosciszewski et al. (2018). In these conditions, purified astrocytes did not achieve a high NF-κB activation level (nuclear p65 localization without cytoplasmic p65 staining) following HMGB-1 exposure (Figure 4F). They rather became mildly activated, showing similar intensity in nuclear and cytoplasmic p65 staining, while inactive cells showed negative nuclear p65 staining (Figure 4F). Conversely, the presence of microglia facilitated astrocytic NF-κB activation, since highly NF-κB -activated astrocytes after HMGB-1 exposure were only observed in the presence of microglia (Figure 4G). We conclude that microglia facilitates NF-κB activation in astrocytes following HMGB-1 exposure. Then, we wondered if HMGB-1 had a direct effect on microglia. To test that hypothesis, we exposed pure microglial cultures to HMGB-1 and analyzed NF-κB activation, microglial cell morphology and the expression of TREM-2 as a marker of the M2 anti-inflammatory phenotype. HMGB-1 exposure induced NF-κB activation in microglial cultures, but to a lesser extent than the activation level observed in response to LPS exposure used as a pro-inflammatory control stimulus (Figure 4H). On the other hand, neither the morphology nor TREM-2 expression was significantly affected by HMGB-1 exposure (Figures 4I,J). We conclude that HMGB-1 activates an NF-κB dependent pathway in glial cells. While HMGB-1 displays a direct effect on microglia, astrocytes require the presence of microglia to achieve the highest level of NF-κB activation.

### HMGB-1 –Induced NF-κB Activation in Primary Glial Cultures Is TLR2/TLR4/RAGE-Dependent

**FIGURE 5:**
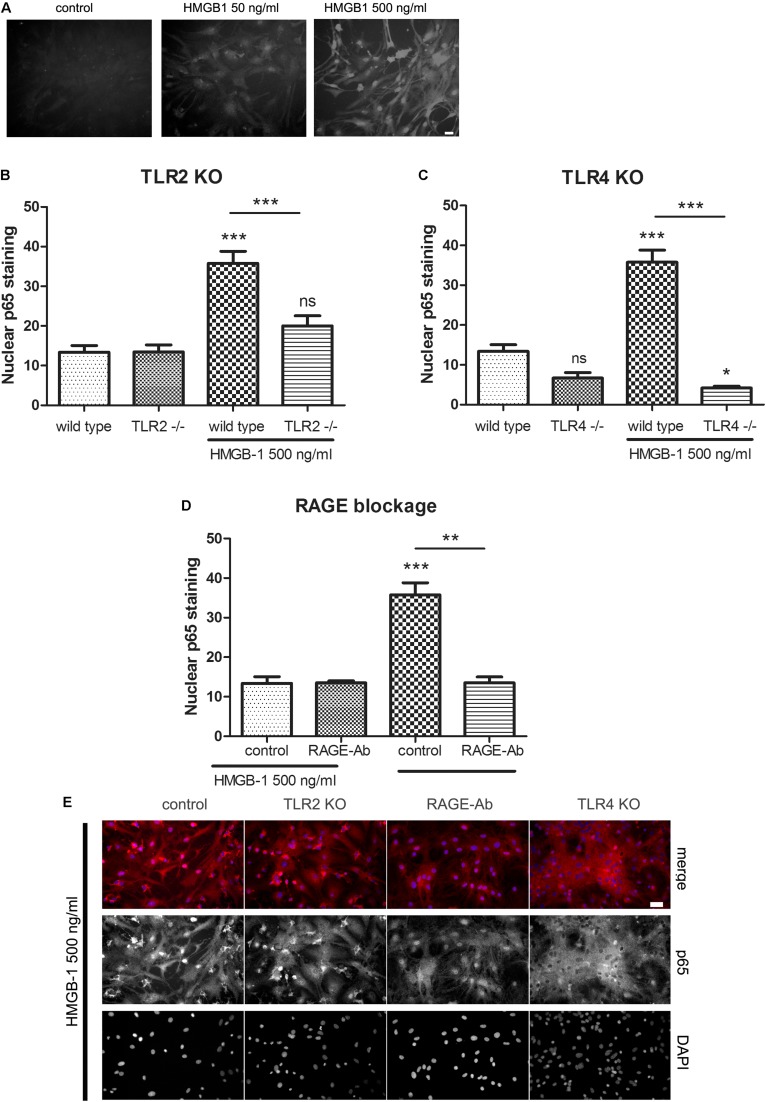
HMGB-1 –induced NF-κB activation in astrocytes depends on the PRR TLR2, TLR4, and RAGE. **(A)** Representative images of glial cell cultures containing approximately 95% astrocytes (star-like cells) and 5% microglia (small rounded cells) exposed to Histidine-tagged recombinant HMGB-1 for 1 h, medium was replaced and immunocytochemistry for the His-tag was performed; bar = 10 μm. **(B–D)** Quantitative results of the p65 NF-κB subunit nuclear localization in glial cell cultures obtained from wild type, TLR2 **(B)**, TLR4 **(C)** knockout mice or incubated with the RAGE neutralizing antibody (1 ug/ml) **(D)**. **(E)** Representative images of p65 NF-κB subunit nuclear localization in glial cell cultures exposed to HMGB-1 and immunostained for p65 (red) and counterstained with nuclear DAPI. Control cultures were wild type glial cell cultures incubated with an unspecific antibody of the same isotype as anti-RAGE; bar = 40 μm. In all cases, glial cultures were exposed to 500 ng/ml HMGB-1 during 3 h. Data show the percentage of GFAP + astrocytes showing nuclear p65 NF-κB subunit localization. Statistical analyses were performed by one way ANOVA and Student Newman–Keuls post-test, with statistical significance represented as ^∗^*p* < 0.05, ^∗∗^*p* < 0.01, and ^∗∗∗^*p* < 0.001. Data presented in the graphs are mean ± SEM from three experiments.

Previous reports have shown that HMGB-1 effects are mediated by different PRR including TLR2, TLR4, and RAGE (reviewed in Tian et al., 2017); however, the reported studies comprised different cell types. As stated above, our results have shown that glial-mediated HMGB-1 detrimental effects on neurons require TLR2 and TLR4 signaling (Figure 3). We then decided to perform a loss-of-function study by analyzing the specific contribution of TLR2, TLR4, and RAGE to the HMGB-1 effects in glial cells. Firstly, we analyzed whether HMGB-1 effectively binds to glial cells *in vitro* by exposing cultures to human recombinant His-tagged HMGB-1, subsequent washing and revealing the bound protein with an anti-His tag specific antibody avoiding the permeabilization step. As shown in Figure 5A, immunofluorescence showed a dose-dependent increase in the HMGB-1 binding to microglia and astrocytes, demonstrating that HMGB-1 binds to glial cells. To analyze the signaling pathways activated by HMGB-1 in glial cells and the requirement of TLR2 and TLR4 for downstream HMGB-1-induced NF-κB activation, we exposed mixed glial cultures from TLR4 or TLR2 knockout animals to HMGB-1. As shown in Figures 5B,C, the HMGB-1 effect on astrocytes from mixed glial cultures is TLR2- and TLR4-dependent, since a decreased NF-κB activity was observed in the knock out cultures exposed to HMGB-1. RAGE dependence was established by blocking RAGE with neutralizing antibodies, and Figure 5D shows that NF-κB activation in glial cells is abolished by pre-incubation with these blocking immunoglobulins. In Figure 5E, representative images of the p65 NF-κB subunit nuclear localization patterns in the different loss-of-function paradigms are shown. We conclude that HMGB-1 –induced NF-κB activation in glial cells can be mediated by either TLR2, TLR4, or RAGE-dependent signaling.

### *In vivo* HMGB-1 Blockage With Glycyrrhizin Reduces Reactive Gliosis and Neuronal Degeneration in a Model of TLE

**FIGURE 6:**
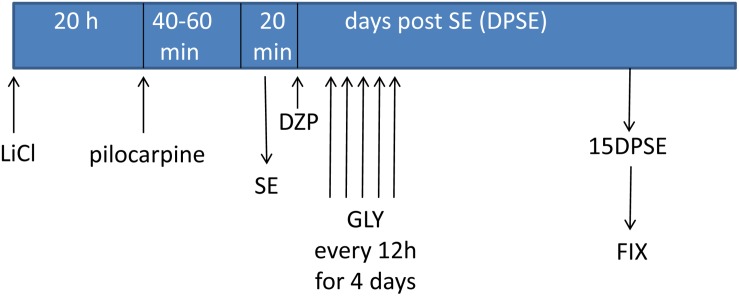
Schematic representation of the *in vivo* treatment with HMGB-1 antagonist glycyrrhizin after pilocarpine-induced status epilepticus (SE).

**FIGURE 7:**
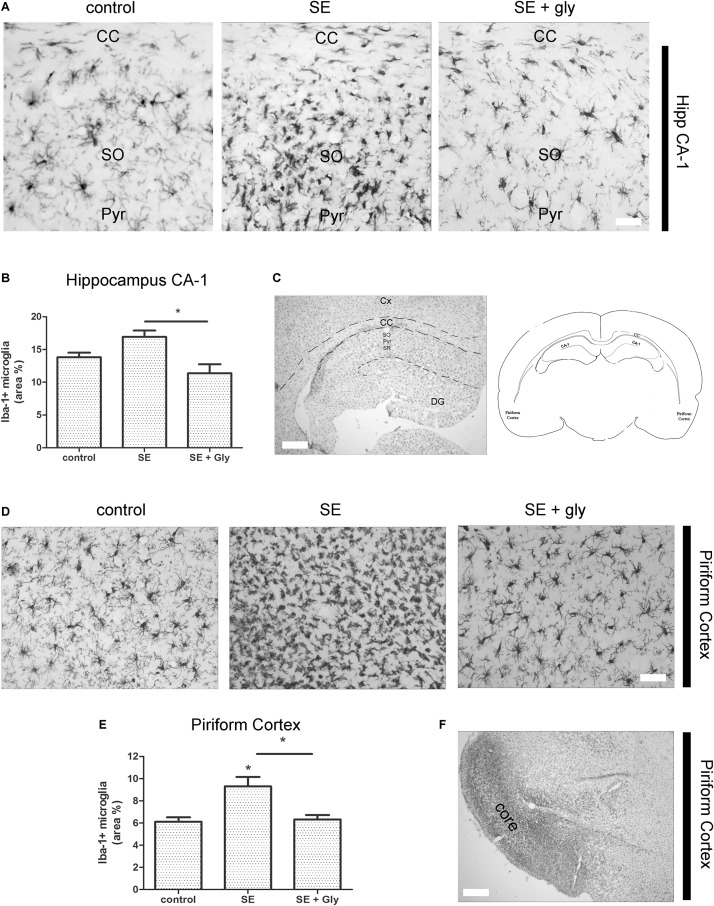
HMGB-1 antagonist glycyrrhizin reduces reactive microgliosis after pilocarpine-induced SE. Rats were exposed to pilocarpine-induced SE, treated with glycyrrhizin or vehicle for 4 days and analyzed after 15 days. **(A)** Representative images of Iba-1-immunostained microgliocytes in the CA-1 hippocampal area showing hippocampal Stratum Oriens (SO), hippocampal pyramidal layer (Pyr), and also the Corpus Callosum (CC). Note the increased microglial cell abundance in SE exposed animals and the decrease in microglial cell abundance in SE glycyrrhizin-treated animals. Scale bar: 15 μm. **(B)** Quantitative analysis of the Iba-1 + microglial cell abundance hippocampus of control, SE and SE animals treated with glycyrrhizin. **(C)** Low magnification of the Iba-1-immunostained hippocampus to visualize the different regions (scale bar: 350 μm) and the schematic representation of the analyzed areas in a coronal rat brain section. Cx, Brain cortex; CC, Corpus Callosum; DG, Dentate Gyrus; CA-1, Hippocampal CA-1 area; SR, Stratum Radiatum; SO, Stratum Oriens; Pyr, Pyramidal neurons. **(D)** Representative images of Iba-1-immunostained microgliocytes in the piriform cortex. Scale bar = 30 μm. Note that necrotic core has been colonized by microgliocytes and thus it is not distinguished in these images but noticeable in the low magnification image **(F)**. **(E)** Quantitative analysis of Iba-1-immunostained cell area in the piriform cortex and hippocampus of control, SE and SE animals treated with glycyrrhizin. Statistical analyses were performed by one way ANOVA and Student Newman–Keuls post-test, with statistical significance represented as ^∗^*p* < 0.05, ^∗∗^*p* < 0.01, and ^∗∗∗^*p* < 0.001. Control animals were exposed to lithium chloride and saline was used as vehicle. The number of animals were *n* = 6 per experimental group.

**FIGURE 8:**
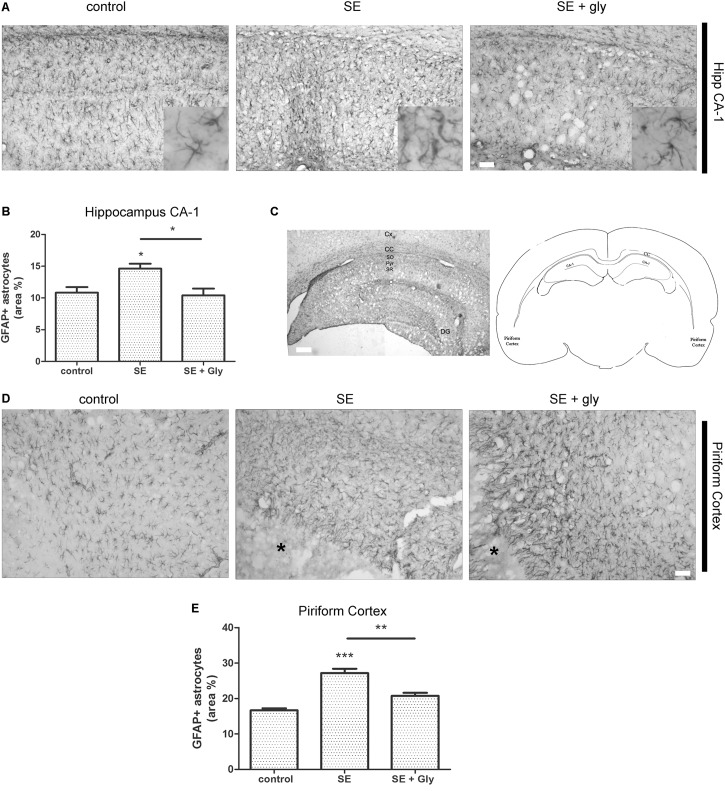
HMGB-1 antagonist glycyrrhizin reduces reactive astrogliosis after pilocarpine-induced SE. Rats were exposed to pilocarpine-induced SE, treated with glycyrrhizin or vehicle for 4 days and analyzed after 15 days. **(A)** Representative images of GFAP-immunostained astrocytes showing the CA-1 hippocampal area. The insets depict astroglial cell morphology in detail. Note the increased astroglial hypertrophy with enlarged projection and increased soma size in SE-exposed animals and the decreased hypertrophy in SE glycyrrhizin-treated animals. Scale bar: 30 μm. **(B)** Quantitative analysis of the GFAP + astroglial cell morphology in the hippocampus of control, SE and SE animals treated with glycyrrhizin. **(C)** Low magnification of the GFAP-immunostained hippocampus to visualize the different regions (scale bar: 300 μm) and a schematic representation of the analyzed areas in a coronal rat brain section. Cx, Brain cortex; CC, Corpus Callosum; DG, Dentate Gyrus; CA-1, Hippocampal CA-1 area; SR, Stratum Radiatum; SO, Stratum Oriens; Pyr, Pyramidal neurons. **(D)** Representative images of GFAP-immunostained astrocytes in the piriform cortex. Note the necrotic core (^∗^) that is characteristic of SE-exposed animals surrounded by highly hypertrophied astrocytes. Scale bar = 35 μm. **(E)** Quantitative analysis of GFAP-immunostained cell area in the piriform cortex and hippocampus of control, SE and SE animals treated with glycyrrhizin. Statistical analyses were performed by one way ANOVA and Student Newman–Keuls post-test, with statistical significance represented as ^∗^*p* < 0.05, ^∗∗^*p* < 0.01, and ^∗∗∗^*p* < 0.001. Control animals were exposed to lithium chloride and saline was used as vehicle. The number of animals were *n* = 6 per experimental group.

**FIGURE 9:**
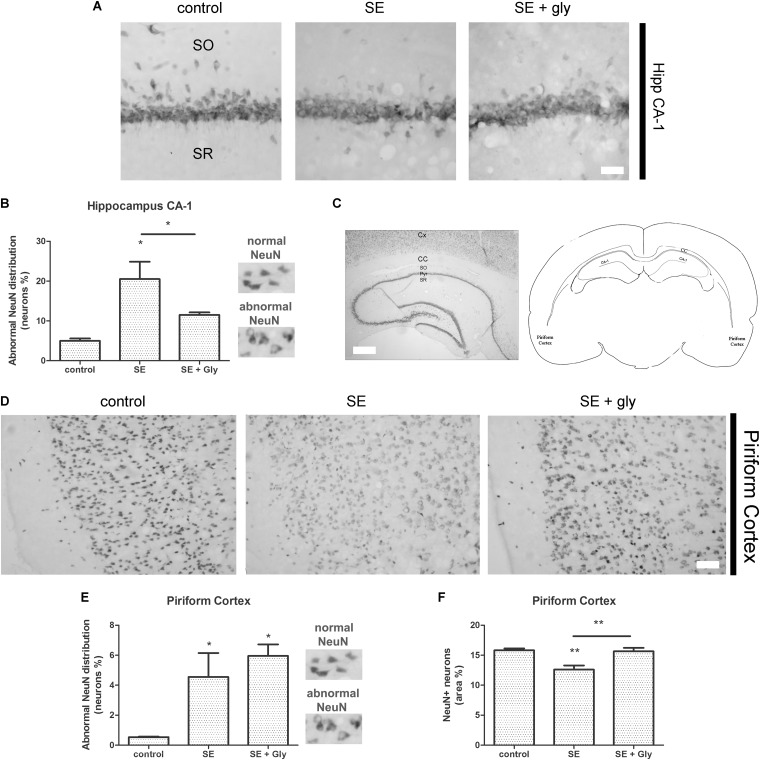
HMGB-1 antagonist glycyrrhizin improves neuronal survival after pilocarpine-induced SE. Rats were exposed to pilocarpine-induced SE, treated with glycyrrhizin or vehicle for 4 days and analyzed after 15 days. **(A)** Representative images of NeuN immunostaining that labels the CA-1 hippocampal pyramidal neurons. Stratum Oriens (SO) and Stratum Radiatum (SR) are also shown. Note the decreased NeuN immunostaining and NeuN relocalization to the cytoplasm, both features of neurodegeneration, in SE-exposed animals and the partial recovery induced by glycyrrhizin treatment. Scale bar: 28 μm. **(B)** Quantitative analysis of NeuN-immunostained neurons showing atypical NeuN localization in the cytoplasm in the CA-1 pyramidal cell layer of control, SE and SE animals treated with glycyrrhizin. **(C)** Low magnification of the NeuN-immunostained hippocampus to visualize the different regions and the typical NeuN labeling of hippocampal neurons. Scale bar: 350 μm. Cx, Brain cortex; CC, Corpus Callosum; SO, Stratum Oriens; Pyr, Pyramidal cell layer; SR, Stratum Radiatum; CA-1, Hippocampal CA-1 area. The scheme shows the localization of the analyzed areas in a coronal rat brain section **(D)** Representative images of NeuN-immunostained neurons in the piriform cortex, images were taken in caudal position to the necrotic core. The absence of NeuN immunostaining in the brain cortical layer I (*moleculare*), scale bar: 40 μm. **(E)** Quantitative analysis of the atypical NeuN-immunostained neurons showing the NeuN redistribution to the cytoplasm in the piriform cortex of control, SE and SE animals treated with glycyrrhizin. **(F)** Quantitative analysis of the total area of NeuN-immunostained neurons in the piriform cortex of control, SE and SE animals treated with glycyrrhizin. Note that glycyrrhizin treatment did not prevent the atypical NeuN localization but significantly reduced neuronal loss induced by SE. Statistical analyses were performed by one way ANOVA and Student Newman–Keuls post-test, with statistical significance represented as ^∗^*p* < 0.05, ^∗∗^*p* < 0.01, and ^∗∗∗^*p* < 0.001. The number of animals were *n* = 6 per experimental group.

Having established that HMGB-1 activates PRR-dependent signaling pathways in glial cells and that it is detrimental for neuronal survival, we decided to analyze the neuronal and glial effects of HMGB-1 blockage after the SE induced by lithium-pilocarpine administration in rats. The lithium-pilocarpine model of TLE is a well-established epilepsy paradigm that reproduces most of the features of human TLE, by inducing a precipitating event (SE) followed by a silent epileptogenic period, and subsequently developing spontaneous epileptic seizures (reviewed in Curia et al., 2008). SE induced by lithium-pilocarpine treatment produces sustained reactive gliosis and neurodegeneration in piriform cortex and hippocampus, which are hypothesized to be essential for epileptogenesis (Rossi et al., 2013, 2017). Here, animals received the HMGB-1 blocking drug glycyrrhizin twice a day for 4 days, after developing SE (4DPSE) by lithium-pilocarpine administration and were analyzed after 15 days (15DPSE) (Figure 6). As shown in Figure 7, animals treated with glycyrrhizin showed a significative reduction in reactive microgliosis compared with the SE exposed animals that received vehicle. Early treatment with glycyrrhizin drastically reduced reactive Iba-1 + microglia in hippocampal CA-1 (Figures 7A–C) and piriform cortex (Figures 7D,E), although glycyrrhicin did not prevent the formation of a necrotic core in the piriform cortex (Figure 7F). Reactive astrocytes overexpress GFAP showing evident signs of hypertrophy, enlarged projections and soma size. As shown in Figure 8, glycyrrhizin early treatment also attenuated reactive gliosis in the hippocampal CA1 region (Figures 8A–C) and piriform cortex (Figures 8D,E). In addition, glycyrrhizin treatment prevented neuronal alterations evidenced by atypical NeuN mobilization from the nucleus to the cytoplasm, in the pyramidal cell layer of hippocampal CA-1 area (Figures 9A–C). However, glycyrrhizin treatment was not able to prevent NeuN alypical sub-cellular distribution in the piriform cortical neurons, but successfully reduced neuronal loss in that brain area (Figures 9D–F). It should be noted that NeuN mobilization to the neuronal cytoplasm is an early marker of reversible neuronal alterations, while its disappearance evidence neuronal loss (Robertson et al., 2006; Angelo et al., 2014). We conclude that HMGB-1 blockage immediately after SE, during the silent period that follows this IPE, is beneficial to reduce reactive gliosis and to improve neuronal survival.

## Discussion

HMGB-1 is a prototypical DAMP released after cell injury and capable of activating innate immune responses. In the CNS, HMGB-1 was shown to be released after different types of acute injury (Muhammad et al., 2008; Qiu et al., 2008, 2010; Gao et al., 2012, 2018; Laird et al., 2014; Sun et al., 2014; Haruma et al., 2016; Parker et al., 2017) and after SE (Fu et al., 2017). Extracellular HMGB-1 interacts with TLR2, TLR4, and RAGE to induce innate immunity activation and neuroinflammation within the CNS (Qiu et al., 2010; Yang et al., 2010; Weber et al., 2015; reviewed in Tian et al., 2017). Once released into the brain parenchyma, HMGB-1 can reach the blood, where brain-derived HMGB1 can be redox modified in the circulation; the oxidized form acts as a cytokine targeting peripheral organs, specifically bone marrow and spleen, to recruit myeloid cells and activate peripheral immune cells (Liesz et al., 2015; Singh et al., 2016). Activated immune competent cells also actively release an acetylated form of HMGB-1, probably as a result of maturation in the periphery (Venereau et al., 2016; Singh et al., 2016).

In epilepsy studies, HMGB-1 has gathered increasing attention because it has been hypothesized that it may play a role in epileptogenesis, being both a pharmacological target and a biomarker for the silent phase of the disease (reviewed in Paudel et al., 2018). Animal models of acute and chronic seizures have shown that HMGB-1 receptors are expressed after experimental seizures (Maroso et al., 2010; Rossi et al., 2017) and that HMGB-1/TLR4 signaling plays a role in generating and perpetuating seizures by modifying ionotropic glutamatergic subunit NR2B phosphorylation (Maroso et al., 2010). Human tissue from epileptic lesions has shown overexpression of HMGB-1 and the proposed binding receptors TLR2, TLR4, and RAGE (Zurolo et al., 2011).

Our present results support the assumption that HMGB-1 dependent signaling pathways appear to be centrally involved in the well-characterized initial neuroinflammation and neurodegeneration that follow the IPE (Rossi et al., 2013, 2017). HMGB-1 is released after SE (Fu et al., 2017) and we here propose that, acting on astrocytes and microglia, HMGB-1 activates TLR2/TLR4/RAGE signaling pathways that facilitate neurodegeneration. Undoubtedly, our results also support that HMGB-1-dependent signaling pathways are also likely to be involved in other acute injuries in the CNS such as TBI and ischemia where HMGB-1 was shown to be released from the necrotic core (Muhammad et al., 2008; Kim et al., 2018).

*In vitro* experiments using hippocampal mixed cultures containing glial cells and neurons showed the ability of HMGB-1 to induce neurite retraction and reactive gliosis. The dose-response studies show that neurodegenerative HMGB-1 effects correlate with reactive gliosis. This neurotoxic effect is abolished when neurons are seeded in absence of microglia and astrocytes. These observations remarkably show that glial cells are required for the detrimental HMGB-1 effects on neuronal survival to become evident. Previous reports have proposed that neurodegenerative HMGB-1 effects after SE and TBI are due to HMGB-1-induced alterations in blood-brain barrier permeability (Laird et al., 2014; Fu et al., 2017). We here report that exogenously applied HMGB-1 is able to induce neuronal degeneration when glial cells are present in the culture, showing that HMGB-1 requires glial cells to promote neurodegeneration.

As a DAMP, HMGB-1 has the ability of activating PRR, which are innate immunity receptors that are also activated by PAMP. By comparing HMGB-1 with the prototypical pro-inflammatory PAMP LPS, we here show that both HMGB-1 or LPS-treated glia induces neuronal death. Signaling pathways activated by HMGB-1 in different cell types, including the professional immune cells, are triggered by engaging the classical innate immunity receptors RAGE, TLR2, and TLR4 (Pedrazzi et al., 2007; Choi et al., 2017; Paudel et al., 2018). By combining glial cell cultures obtained from TLR2 knockout mice brains and TLR4 pharmacological blockade with the chemical inhibitor VGX-1027, we demonstrate here that detrimental HMGB-1 effects on neurons are mediated by glial TLR2 and TLR4. Upon ligand binding, TLR2 or TLR4 initiate a well-characterized signal transduction pathway, that leads to the activation of NF-κB and expression of pro-inflammatory target genes (reviewed in Crack and Bray, 2007; Tajalli-Nezhad et al., 2018). By using glial cell cultures containing astrocytes and microglia, we here show that both cell types exhibit a significant NF-κB activation following HMGB-1 exposure, and that this activation is TLR2/TLR4/RAGE-dependent. However, astroglial enriched cultures containing less than 1% microglia showed a reduced NF-κB activation, thus suggesting that microglial cells are necessary to achieve a significant level of NF-κB activation in astrocytes after HMGB-1 exposure. Microglial cultures lacking astrocytes, on the other hand, still responded to HMGB-1 but to a lesser extent than to the prototypical PAMP LPS, and failed to show the phenotypical switch to the amoeboid activated state, or to change the expression of the classical M2 phenotype marker TREM-2. HMGB-1-induced AQP4 expression was previously shown to depend on microglia-astroglial interaction through soluble mediators (Ohnishi et al., 2014). Moreover, Gao et al. (2011) have shown that dopaminergic neurodegeneration in experimental Parkinson Disease requires HMGB-1-activated microglia and downstream NF-κB signaling (Gao et al., 2011). HMGB-1 exposure also seems to induce pro-inflammatory priming in microglial cells of aged brains (Fonken et al., 2016) and the microglial inhibitor named minocicline reduces reactive microgliosis and HMGB-1 release by activated glia (Hayakawa et al., 2008) Stroke. In this scenario, our results support the notion that microglial-astroglial interaction is required for glial cells to fully respond to HMGB-1, and that this interaction is also required for the HMGB-1 neurodegenerative effects to become evident, since HMGB-1 was unable to reduce neuronal survival in primary cultures lacking glial cells. In addition, our findings in reconstituted cultures that prevent cell contact support the notion that soluble glial cell-derived neurotoxic mediators released upon HMGB-1 stimulation are those able to induce neuronal degeneration without requiring cell contact to exert their effect. The obvious candidate molecules to mediate this effect are classical proinflammatory cytokines such as IL-1β and TNF-α as well as the complement molecule C1q. All these molecules have shown to facilitate astroglial polarization to the proinflammatory-neurodegenerative phenotype (Liddelow et al., 2017). However, more complex cell-to-cell communication pathways like extracellular vesicles can not be ruled out. This area requires further studies in the near future.

Upon binding to target receptors, extracellular HMGB-1 behaves as a typical DAMP, activating PRR-dependent signaling pathways (reviewed in Paudel et al., 2018). We here demonstrated that HMGB-1 induces the translocation of NF-κB to the glial cell nucleus in a TLR2-, TLR4-, and RAGE-dependent manner. The NF-κB-dependent pro-inflammatory responses are probably centrally involved in the neurodegeneration induced by HMGB-1-activated glial cells. Taken together, our results show a novel microglial-astroglial cooperation required for the DAMP HMGB-1 to induce neurodegeneration. This cellular interaction reflects the astroglial engagement in innate immunity and is likely to be a common pathway in brain injury.

A growing body of evidence shows the beneficial role of interfering with HMGB-1 effects after brain injury. HMGB-1 blockade using neutralizing antibodies has been repeatedly shown to be beneficial for brain ischemia and TBI (Okuma et al., 2012; Wang C. et al., 2016), to prevent BBB disruption in a model of Alzheimer’s disease (Festoff et al., 2016), reduce 6 hydroxy-dopamine induced neuronal death (Sasaki et al., 2016) and to reduce neuroinflammation and neurocognitive dysfunction in the aged brain (Fonken et al., 2016; Terrando et al., 2016).

The natural molecule glycyrrhizin, a component from the liquorice root, has also been tested to block HMGB-1 effects since it directly binds to HMGB-1 preventing its interaction with ligand receptors (Okuma et al., 2014). Glycyrrhizin administration was shown to reduce dopamine neuronal death in experimental models of Parkinson’s disease (Qi et al., 2015; Santoro et al., 2016). Glycyrrhizin treatment after experimental stroke was effective in reducing infarction (Kim et al., 2012) and neuroinflammation (Xiong et al., 2016), ameliorated intracerebral hemorrhage-induced edema and neuronal loss (Ohnishi et al., 2011), reduced isofluorane-induced neuronal death in neonatal brains (Wang W. et al., 2016) and diminished motor deficits as well as neuroinflammation after experimental TBI (Okuma et al., 2014).

It has been reported that a transient induction of HMGB-1 release occurs after pilocarpine-induced seizures (Fu et al., 2017) in a striking similarity to the HMGB-1 release previously reported in other acute brain injuries (Muhammad et al., 2008; Kim et al., 2018). Accordingly, anti-HMGB-1 antibody administration after seizures reduces neuronal death, acute cytokine release, and astroglial and microglial reactivity in the acute time frame (Fu et al., 2017). In addition, glycyrrhizin administration 30 min before initiating kainic acid-induced seizures in mice suppresses HMGB-1 release (Luo et al., 2014), and consequently reduces reactive gliosis and neuronal death (Luo et al., 2013, 2014). Very recently, Li et al. (2018) have shown that glycyrrhizin treatment ameliorates acute hippocampal neuronal damage and reduces BBB disruption after lithium-pilocarpine treatment (Li et al., 2018). Together, this evidence points toward a main role of HMGB-1 released in the early stages that follow an IPE. In agreement with this, and extending these previous findings, we here show that glycyrrhizin administrated 30 min after pilocarpine-induced seizures partially protects neurons and reduces reactive gliosis in the long term. Thus, having in mind our *in vitro* and *in vivo* mechanistic findings, we propose that early interference with HMGB-1 released by seizure-damaged neurons during the initial latency period that follows the IPE is able to reduce glial conversion to the pro-inflammatory-neurodegenerative phenotype, and that this interference produces beneficial long-lasting effects in neuronal survival and neuroinflammation. Thus, either HMGB-1 blockage or TLR4/TLR2/RAGE antagonist molecules would be able to reduce neuroinflammation and neurodegeneration, phenomena which are proposed as key early steps in epileptogenesis. Taking together our present results with the available previous published data, HMGB-1 and its receptors emerge as a tempting pathway to target in order to change the development of epileptogenesis and probably the natural history of epilepsy as a disease. Lastly, the novel astroglial-microglial cooperation required for HMGB-1 to produce its effects on neuronal survival described here, emerges as a potentially shared common pathway in the acute injury in the CNS. Thus our findings could be extended to several other types of acute brain injury, most notably to TBI and brain ischemia where HMGB-1 has been shown to play a major role.

## Data Availability

All datasets generated for this study are included in the manuscript and/or the supplementary files.

## Ethics Statement

All procedures involving animals and their care were conducted in accordance with our institutional guidelines, which comply with the NIH guidelines for the Care and Use of Laboratory Animals, the principles presented in the Guidelines for the Use of Animals in Neuroscience Research by the Society for Neuroscience, the ARRIVE guidelines and were approved by the CICUAL committee of the School of Medicine, University of Buenos Aires (Res. Nr. 1278/2012). All efforts were made to minimize animal suffering and to reduce the number of animal used.

## Author Contributions

AJR conceived the project, designed the experiments, analyzed the data, and wrote the manuscript. FG, ARR, AR, VM, and AV designed the experiments, discussed results and revised the manuscript. GR, VC, JL, JA, MC, and ARR did the experiments and analyzed the data.

## Conflict of Interest Statement

The authors declare that the research was conducted in the absence of any commercial or financial relationships that could be construed as a potential conflict of interest.
